# Enhancing performance of berseem clover genotypes with better harvesting management through farmers’ participatory research at smallholder farms in Punjab

**DOI:** 10.1038/s41598-020-60503-7

**Published:** 2020-02-26

**Authors:** M. S. Tufail, G. L. Krebs, A. Southwell, J. W. Piltz, M. R. Norton, P. C. Wynn

**Affiliations:** 1 Department of Agronomy, University of Agriculture Faisalabad, Sub-Campus Depalpur (Okara), Punjab 56300 Pakistan; 2 Graham Centre for Agricultural Innovation, Charles Sturt University, Wagga Wagga, 2650 New South Wales Australia; 3 School of Animal and Veterinary Sciences, Charles Sturt University, Wagga Wagga, New South Wales 2678 Australia; 4 New South Wales’ Department of Primary Industries, Pine Gully Road, Wagga Wagga, New South Wales 2650 Australia

**Keywords:** Plant breeding, Plant evolution

## Abstract

A field study was conducted on smallholder farmer fields between 2012 to 2014 to evaluate the performance of cv. Agaitti Berseem-2002, against local landraces exchanged between farmers (LBF1) or available from local markets (LBM1). The effects of genotype and harvesting regimen on forage production, quality and seed production were evaluated. Significant differences (*P* < 0.05) among genotypes and cutting treatments were recorded for forage and seed yields, and forage quality across all research sites in both years. Maximum cumulative fresh forage (89.7 t/ha) and dry matter (DM; 13.4 t/ha) yields were obtained with Agaitti Berseem-2002 when harvesting occurred five times over the season. However, maximum seed yield (1048 kg/ha) with higher 1000-seed weight (3.63 g) were obtained if forage was only harvested three times and the crop then left for seed set. Agaitti Berseem-2002 also produced forage with the higher crude protein content (27%), DM digestibility (69%), digestible organic matter (DM basis; 65%) and metabolizable energy content (10%) compared to the local landraces (LBF1 and LBM1). Therefore, the harvesting regimen for greatest economic return which produced optimum fresh and DM forage yields of highest nutritive values and maximum seed yield, were comprised of taking three forage cuts (at 65, 110 and 150 days after sowing) prior to seed harvest.

## Introduction

In Pakistan, livestock production in mixed farming systems typically suffers from both limited quantity and quality of forage. Pakistan’s annual livestock feed requirements are met through a combination of green forages (51%), crop residues (38%), grazing (3%) and concentrates (2%). Collectively, these animals require 11 million tonnes (Mt) of crude protein (CP) and 90 Mt of total digestible nutrients (TDN) annually, but only receive 7 and 69 Mt per year of these nutrients, respectively^1^.

Forage deficits may result from a range of factors including land scarcity, soil nutrient imbalance, water deficiency, poorly adapted germplasm and extreme weather events^2^. A lack of farmer knowledge of forage production, utilisation and conservation techniques may also contribute to the problem^3^. Smallholder farmers perceive that these feed gaps could be filled by increasing forage production per unit area, through the use of improved varieties, appropriate agronomic practices and timely availability of required inputs^4^. Forage production is also often constrained by the availability of quality seed^3^. Consequently, farmers often have to produce their own seed, even if it is inferior.

Berseem clover is considered as a strategic forage crop in the sustainability of agriculture production systems due to its low input requirement and restorative nature^5^. Berseem growers have mostly planted this as a dual purpose crop for both forage and seed production^6^. There are different yield components attributed towards berseem crop yield but the genotype and harvesting management are the key factors in obtaining quality forage and seed yields^7,8^. However, scientific information is still lacking regarding the effects of genotypes, and different forage cutting regimens on the forage and seed yields as well as on the nutritive values of berseem forage. Therefore, knowledge of the dry matter accumulation over time in the berseem plant is crucial and can determine the strategy for harvesting of different genotypes.

In Pakistan berseem clover (*Trifolium alexandrinum* L.) is commonly a multi-cut forage crop, belonging to the Miscawi crown branching type able to produce 5–6 forage cuts per growing season^9^ with seed yield being dependent on plant regrowth following the last forage harvest. Adoption of improved varieties by smallholder farmers largely depends on the varietal information they acquire, and involvement of farmers in the evaluation process expedites this^3^. Traditionally, varietal selection has been carried out using the conventional top-down model rather than the model of participatory varietal selection by farmers. The participatory approach has been shown to be a workable strategy in quality forage and seed production, and dissemination of the best improved varieties in Punjab, Pakistan^10^. The most appropriate model of participatory varietal selection and evaluation is where the researcher is responsible for germplasm selection, and cultivar development^11^. The researcher and farmer then work together to evaluate the chosen cultivar at the farm level. Moreover, the participatory research approach is demand driven and has benefits related to the adoption of new improved varieties.

The aim of the present study was to determine which berseem clover genotype (seed source) and cutting regimen gave the best results in terms of quality forage and seed production at farm level. The study was designed to involve the smallholder farmers through participatory varietal selection and evaluation, to promote knowledge and adoption of best cutting practices and varieties for berseem clover production.

## Materials and Methods

Two experiments were conducted over the 2012/13 and 2013/14 growing seasons in Kasur (31.1165°N, 74.4494°E, altitude 218 m) and Okara (30.8090°N, 73.4508°E, altitude 170 m) districts in Punjab province, Pakistan, which are located in the semi-arid zone^12^.

### Year 1 experiment

The 2012/13 experiment was conducted over nine sites each with three genotypes of berseem seed and four cutting treatments in a randomised complete block design. Seed genotypes were: (1) a landrace produced by local farmers (LBF1) retained on-farm, (2) a landrace (LBM1) sold locally in the agricultural market and (3) Agaitti Berseem-2002, an improved variety bred at the Fodder Research Institute (FRI), Sargodha-Pakistan. One lot of each seed was purchased and used across all the research sites in both years. The four cutting treatments were: two forage cuts [at 65 and 110 days after sowing (DAS)] prior to seed harvest (T1), three forage cuts (at 65, 110 and 150 DAS) prior to seed harvest (T2), four forage cuts (at 65, 110, 150 and 180 DAS) prior to seed harvest (T3), and five forage cuts (at 65, 110, 150, 180 and 210 DAS) prior to seed harvest (T4).

The sites were on eight farms in the Kasur and Okara districts together with the University of Veterinary and Animal Sciences (UVAS) Research Farm, at Ravi-campus Pattoki, Kasur district, Punjab. Each of the trials sites was treated as a replicate in order to address soil heterogeneity, irrigation sources and farmers’ cultural practices. Eight smallholder farmers registered with the Australian Centre for International Agricultural Research (ACIAR) dairy extension project (LPS/2010/007) entitled, “Improving dairy value chains in Pakistan through improved extension services”, were selected from the region based on having the appropriate skills and knowledge of berseem clover seed production. Each research participant (farmer) was provided with an information sheet, consent form, and briefed about the participatory research and their role before the start of the on-farm experimentation, with the prior approval of Charles Sturt University’s Human Research Ethics Committee (Protocol # 416/2012/12). Informed consent had been obtained from all the study participants about their involvement in the participatory research (including trial experiments).

Land preparation included levelling of the seedbed and application of urea (46% N), di-ammonium phosphate (18% N and 46% P_2_O_5_) and muriate of potash (60% K_2_O) fertilizers at 20, 150 and 50 kg/ha, respectively; to achieve the recommended application of 20, 60 and 30 kg/ha of nitrogen (N), phosphorus (P) and potassium (K), respectively. All the fertilizers were broadcast by hand and incorporated into the soil during ploughing prior to sowing. A pre-sowing irrigation was then applied. The seed was inoculated with *Rhizobium trifolii* immediately prior to sowing which occurred during the 2^nd^ week of October^13^. Plot size was 3 m × 7 m (21 m^2^). The crop was cut to 5 cm above ground level at each harvest. Seed harvest occurred in the last weeks of February, March, April and May, for treatments T1, T2, T3 and T4, respectively.

### Year 2 experiment

The experiment in the 2013/14 growing season involved five farmers (two from Kasur and three from Okara) who had also participated in the previous year’s on-farm trials. The best performing variety (Agaitti Berseem-2002) and cutting treatments (T2 and T3) in the first year of trial were sown again on each farm. This was due to the need to provide farmers with the opportunity to produce useable/saleable volumes of seed in the second year to ensure a financial return for involvement in the study and thus their financial security. At each site there were four 0.1 ha plots allowing replication of the two cutting treatments with each site treated as a replicate. The overall design was a randomised complete block with two cutting treatments and five replicate sites. To test for seasonal variation between genotypes, the original treatments used in the 2012/13 season were continued (T1 to T4) in the second year (2013/14) on the UVAS field site. Land preparation, sowing and harvest practices, and data collection and analyzes were as per the previous year’s trial.

### Harvesting and sample collection

At each harvest three × 1 m^2^ quadrats were cut from each plot and fresh weight determined. Plant height (average of 10 plants per quadrat), and number of stems were recorded. A composite 1.0 kg sample from each plot was oven dried (Hot Air oven/T1-OV-H-250, Technology International, Faisalabad-Pakistan) at 70 °C for 72 h to determine dry matter (DM) content and calculate DM yield (t/ha).

A second sample (of 1.0 kg) was dried at 60 °C for 48 h prior to grinding first through a 5 mm and then through a 1 mm screen, and a 150 g sub-sample of this material was dried at 80 °C for 24 h to determine neutral detergent fiber (NDF), acid detergent fiber (ADF) and CP contents as well as DM digestibility (DMD), digestible organic matter (OM), digestibility on a DM basis (DOMD), water soluble carbohydrates (WSC) and metabolizable energy (ME) by Near Infrared Reflectance Spectroscopy (NIRS) using a Bruker multi-purpose analyzer (MPA, Bruker Optik GmbH, Ettlingen, Germany) and OPUS software (version 5.1) with calibrations developed by the New South Wales’ Department of Primary Industries’ Feed Quality Service (FQS). The calibrations were developed using the following methods: NDF and ADF were analyzed sequentially^14^ using the filter bag method (Ankom^®^ 200/220 fiber analyzer, ANKOM technology, Macedon, NY, USA), CP as N × 6.25, with N determined using the Dumas combustion method (Leco CNS 2000^®^ analyzer: Leco, St. Joseph, MI, USA), ash by heating a sample in a muffle furnace at 550 °C for 6 h^15,16^, DMD and DOMD by the pepsin cellulase digestibility assay^15^ and ME calculated as [(DMOD × 0.203)-3.001] to develop NIRS machine calibrations^15^.

At the seed harvest, three × 1 m^2^ quadrats were cut from each plot with number of heads per square meter recorded. Ten seed heads were randomly selected from each plot, with the seeds removed, counted and weighed. Seed weight was determined by counting and weighing 1000 seeds and predicted seed yields (kg/ha) were calculated^17^.

### Statistical analyzes

Each parameter was analyzed using the linear mixed model (Asreml) procedure in GenStat^® ^^18^. Seed genotype, cutting treatment and the interaction of these were treated as fixed effects for all parameters, with farm site as the sole random effect. In addition, years were analyzed separately to check for seasonal variation. Least significant differences (LSD) at the 5% level of significance (*P* < 0.05) were used to compare treatment means.

### Ethical approval

All procedures performed in studies involving human participants were in accordance with the ethical standards. The research work was approved by the Charles Sturt University’s Ethics in Human Research Committee (Protocol number 416/2012/12).

## Results

**Figure 1:**
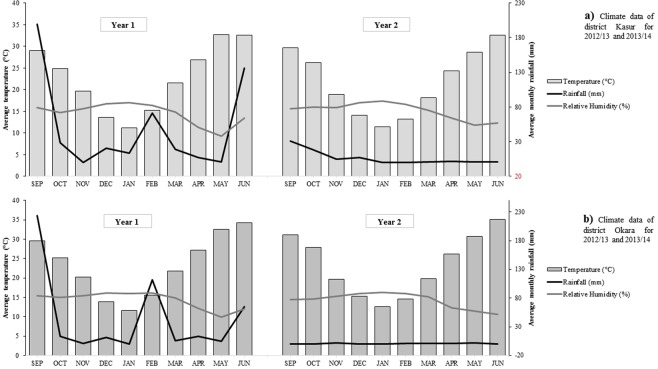
Average rainfall (mm), relative humidity (%) and temperatures (°C) per month during 2012–2013 and 2013–2014 growing seasons (September to June) in Kasur (**a**) and Okara (**b**) districts of Pakistan.

Incident weather at the experimental sites (average rainfall, relative humidity and temperature) is presented in Fig. 1^19^. Both average rainfall and relative humidity were high in season one (2012–13) as compared to season two (2013–14). However, there were few differences in temperature between seasons.

### Varietal selection (Year 1 experiment)

**Figure 2:**
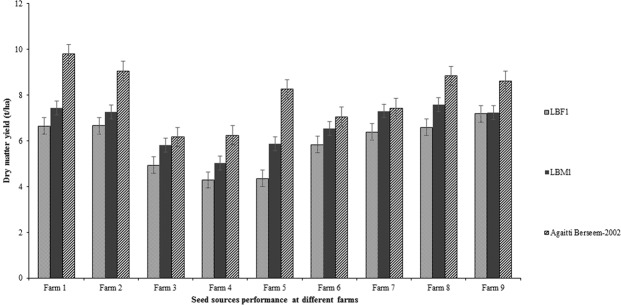
Dry matter forage yield performance of LBF1, LBM1 and Agaitti Berseem-2002 at nine different farms in the districts of Kasur (farm 1–5) and Okara (farm 6–9), Punjab, Pakistan during 2012–2013 growing season.

**Figure 3:**
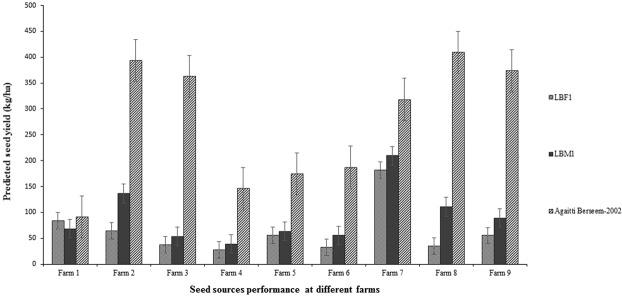
Seed yield performance of LBF1, LBM1 and Agaitti Berseem-2002 at nine different farms in the districts of Kasur (farm 1–5) and Okara (farm 6–9), Punjab, Pakistan during 2012–2013 growing season.

Genotype significantly (*P* < 0.05) affected the DM yield (Fig. 2), seed yield (Fig. 3) and all other forage and seed yield components (stems and seed heads per square meter, plant height, seeds per heads and 1000-seed weight), and forage quality parameters (DMD, DOMD, ME, WSC, CP, NDF, ADF and Ash). Those sites which produced more biomass also produced more seed (*P* < 0.05). The cv. Agaitti Berseem-2002 consistently outperformed both LBF1 and LBM1 in DM and predicted seed yields at all locations.

**Figure 4:**
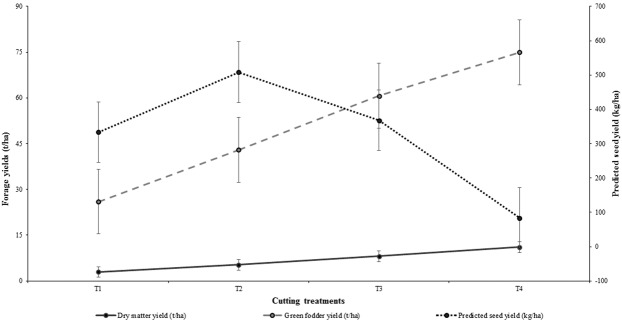
The effect of different cutting regimens on dry matter, green forage and predicted seed yields of berseem clover genotypes across all research sites in the districts of Kasur and Okara, Punjab, Pakistan during 2012–2013 growing season.

The seed yield parameters and predicted seed yield were greater (*P* < 0.05) in district Okara compared to Kasur and there were also significant (*P* < 0.05) differences between farms in both districts. The overall pattern of the forage and seed yields under different cutting treatments is presented in Fig. 4. The maximum total green forage and DM yields were obtained with T4 treatments (with five forage cuts); however, maximum seed yield was recorded with the T2 regimen, having declined under the T3 and T4 cutting treatments. Similar to the forage production, cv. Agaitti Berseem-2002 consistently outperformed both LBF1 and LBM1 at all locations in seed production.

#### Forage and seed yield parameters

The forage and seed yield parameters considered included stem density, plant height, DM yield, seed head density, seeds per head, seed weight and seed yield. The stem density was higher (P < 0.001) in Agaitti Berseem-2002 (397 stems/m^2^) compared to either LBF1 or LBM1 (353 and 352 stems/m^2^, respectively) (Table 1). The interaction between genotype and cutting treatment (P < 0.05) had significant effects on stem number. The stem numbers associated with cutting treatment were 370, 358, 366 and 376 stems/m^2^ for T1, T2, T3 and T4, respectively. The interaction between genotype and cutting treatment was significant (P < 0.05) for plant height (Table 1). Plant height was greater (P < 0.001) for Agaitti Berseem-2002 (26.2 cm) compared to either LBF1 or LBM1 (21.8 cm and 22.4 cm, respectively). Cutting treatment also produced differences (P < 0.001), with T3 and T4 (both 25 cm) yielding taller plants than T1 and T2 plants (21.2 cm and 22.8 cm, respectively). Across all genotypes and cutting treatments, the average plant height was lower (P < 0.001) in Year 1 (22.4 cm) compared to Year 2 (24.5 cm).

**Table 1:** The effect of different genotypes (seed sources; LBF1, LBM1 and Agaitti Berseem-2002) and cutting treatments (T1–T4) on forage and seed yield parameters of berseem clover across all research sites in the Kasur and Okara districts of Punjab, Pakistan during 2012–2013 and 2013–2014 growing seasons.

Parameter	LBF1				LBM1				Agaitti Berseem-2002				*P* value (±SED)
	T_1_	T_2_	T_3_	T_4_	T_1_	T_2_	T_3_	T_4_	T_1_	T_2_	T_3_	T_4_	CT × SS
Number of stems per square meter	343.3^a^	342.5^a^	358.5^ab^	366.7^ab^	332.4^a^	363.0^ab^	351.8^ab^	361.5^ab^	433.5^c^	368.6^ab^	388.5^b^	399.6^bc^	<0.05 ± 19.20
Plant height (cm)	19.3^a^	20.8^b^	23.5^c^	23.5^c^	19.7^ab^	22.0^b^	24.1^c^	23.9^c^	24.5^cd^	25.5^d^	27.3^e^	27.5^e^	<0.05 ± 0.67
Cumulative dry matter yield (t/ha)	2.20^a^	4.42^b^	6.99^cd^	9.39^e^	2.61^a^	5.02^b^	7.80^d^	10.46^f^	4.05^b^	6.59^c^	9.56^ef^	13.37^g^	<0.001 ± 0.51
Number of heads per square meter	177.7^b^	246.9^bc^	308.3^cd^	81.7^a^	221.2^bc^	269.5^c^	282.2^cd^	80.4^a^	358.5^d^	524.2^e^	347.0^cd^	60.6^a^	<0.001 ± 43.01
Number of seeds per head	30.7^b^	34.4^bc^	37.1^c^	11.5^a^	32.7^bc^	38.9^cd^	36.3^bc^	11.0^a^	53.3^de^	56.2^e^	43.8^d^	9.8^a^	<0.001 ± 3.11
1000-seed weight (g)	2.12^b^	2.36^c^	2.39^c^	1.35^a^	1.83^b^	2.52^c^	2.46^c^	1.31^a^	3.49^d^	3.63^d^	3.35^d^	2.01^b^	<0.001 ± 0.15
Predicted seed yield (kg/ha)	148.2^ab^	205.9^ab^	303.7^b^	86.6^a^	182.7^ab^	271.2^b^	282.4^b^	81.1^a^	669.9^c^	1048.0^d^	516.9^c^	83.7^a^	<0.001 ± 87.05

CT: cutting treatment; SS: Seed sources/genotype; SED: Standard error of difference.

As expected, the cumulative DM yield increased with each subsequent cut in all genotypes, averaging 2.9, 5.3, 8.1 and 11.1 t/ha for T1, T2, T3 and T4, respectively. The DM yield of Agaitti Berseem-2002 was higher (P < 0.001) than those of LBF1 or LBM1 for each cutting treatment while LBM1 was significantly higher than LBF1 in the T4 treatment. The maximum cumulative forage DM yield of 13.4 t/ha was achieved using Agaitti Berseem-2002 and five forage cuts (day 65, 110, 150, 180 and 210), while the minimum total forage yield of 2.2 t/ha was with LBF1 and two forage cuts (Table 1). Overall, the average cumulative DM yield of Agaitti Berseem-2002 after taking five forage cuts (T4) was significantly lower (P < 0.001) in Year 1 (10.9 t/ha) compared to Year 2 (15.9 t/ha).

There was a significant interaction (P < 0.001) between the genotype and cutting treatment on the number of seed heads (Table 1). Across all genotypes, the number of seed heads was highest (P < 0.05) in T2 and T3 (347 and 312, respectively), followed by T1 (252) and then T4 (74). Agaitti Berseem-2002 had more (P < 0.05) seed heads than either LMF1 or LBM1 which did not differ (P > 0.05) from each other. The interaction between the genotype and cutting treatment had significant effects (P < 0.001) on the number of seeds produced per head (Table 1). The predicted mean number of seeds per head was higher (P < 0.001) for T2 (43.2) than for T3 and T1 (39 and 38.9, respectively), with T4 significantly lower than all others (10.8). Based on genotype, the predicted mean number of seeds per head was higher (P < 0.001) for forage grown from Agaitti Berseem-2002 (40.8) than forage grown from LBF1 (28.4) or LBM1 (29.7) seeds. Across all genotypes and cutting treatments, the average number of seeds per head was higher (P < 0.001) in Year 1 (35) compared to Year 2 (31). There was a significant interaction (P < 0.001) between genotype and cutting treatment on seed weight (Table 1). Across all genotypes, the predicted mean 1000-seed weight was higher (P < 0.001) for T2 (2.83 g) and T3 (2.73 g) compared to T1 (2.48 g), which was higher (P < 0.001) than that of T4 (1.56 g). Overall, seed weights were higher (P < 0.001) for forage grown from Agaitti Berseem-2002 (3.12 g) than that grown from LBF1 (2.05 g) or LBM1 (2.03 g). Across all genotypes and cutting treatments, the average 1000-seed weight was lower (P < 0.001) in Year 1 (1.91 g) compared to Year 2 (2.89 g).

As shown in Table 1, the interaction between the genotype and cutting treatments had significant effects (*P* < 0.001) on predicted seed yields. In comparing cutting treatments, the predicted seed yields were highest (*P* < 0.001) for T2 and T3 (508 and 368 kg/ha, respectively), although predicted seed yield did not differ (*P* > 0.05) between T3 and T1 (368 and 334 kg/ha, respectively). For all genotypes, predicted seed yield was significantly lower (*P* < 0.001) for T4 (84 kg/ha) compared to other cutting treatments, while T2 and T3 treatments produced significantly (*P* < 0.001) greater seed yields. Based on genotype, predicted seed yield was significantly higher (*P* < 0.001) in Agaitti Berseem-2002 (580 kg/ha) than in LBF1 (186 kg/ha) or LBM1 (204 kg/ha).

#### Forage quality parameters

Forage cuts were taken on ‘cutting days’ 65 (all treatments), 110 (all treatments), 150 (T2, T3 and T4), 180 (T3 and T4) and 210 (T4 only). Significant (*P* < 0.05) differences were found between forage nutritive characteristics due to the cutting treatments (Table 2). The interaction between the genotype and cutting day (*P* < 0.05) had significant effects on DMD and DOMD as well as the CP, NDF and ADF contents. Cutting day also had a significant effect (*P* < 0.001) on ME and the ash content. Across a range of forage quality parameters, the Agaitti Berseem-2002 produced better quality forage compared to LBF1 and LBM1 (Table 2).

**Table 2:** The effect of time of cutting (65, 110, 150, 180 and 210 days after sowing) on forage quality parameters (dry matter basis) of berseem clover genotypes (LBF1, LBM1 and Agaitti Berseem-2002) across all research sites in the Kasur and Okara districts of Punjab, Pakistan during 2012–2013 and 2013–2014 growing seasons.

Parameter	Genotype (seed sources) and cutting time (DAS)															*P* value (±SED)
	LBF1					LBM1					Agaitti Berseem-2002					
	65	110	150	180	210	65	110	150	180	210	65	110	150	180	210	C × SS
DMD (%)	72.1^c^	66.6^ab^	67.1^ab^	65.2^a^	64.6^a^	72.3^c^	66.7^ab^	65.0^a^	65.0^a^	65.0^a^	70.8^bc^	69.9^bc^	66.6^ab^	68.7^b^	65.4^a^	<0.05 ± 1.45
DOMD (%)	67.9^c^	63.3^ab^	63.6^ab^	62.1^a^	61.5^a^	68.0^c^	63.3^ab^	61.9^a^	61.9^a^	61.9^a^	66.8^bc^	66.1^bc^	63.3^ab^	64.9^b^	62.3^a^	<0.05 ± 1.23
ME (MJ/kg)	10.8^e^	9.8^b^	9.9^b^	9.6^a^	9.5^a^	10.8^e^	9.8^b^	9.6^a^	9.6^a^	9.6^a^	10.6^d^	10.4^d^	9.8^b^	10.2^c^	9.6^a^	<0.05 ± 0.10
WSC (%)	2.5	2.4	2.7	2.6	2.5	2.6	2.5	2.5	2.8	2.7	2.6	2.9	2.6	3.2	2.8	ns
CP (%)	29.0^d^	27.9^cd^	27.1^c^	24.7^b^	23.3^ab^	27.8^cd^	27.1^c^	26.2^bc^	23.6^ab^	22.7^a^	27.6^cd^	28.1^cd^	27.2^c^	27.6^cd^	23.3^ab^	<0.001 ± 0.81
NDF (%)	25.5^a^	30.9^c^	29.4^bc^	32.9^cd^	32.5^cd^	26.0^ab^	31.3^cd^	30.5^bc^	33.5^d^	32.9^cd^	28.1^b^	28.2^b^	29.1^bc^	28.3^b^	32.3^cd^	<0.001 ± 1.24
ADF (%)	18.9^a^	21.6^b^	21.9^b^	23.4^c^	24.5^c^	18.8^a^	21.7^b^	23.4^c^	23.7^c^	24.3^c^	19.8^a^	20.1^a^	22.5^bc^	20.2^a^	24.0^c^	<0.001 ± 0.78
Ash (%)	14.5	15.5	15.1	14.6	14.6	14.2	15.1	15.2	14.1	14.2	14.2	14.9	15.1	14.4	14.3	ns

DMD: Dry matter digestibility; DOMD: Digestible organic matter digestibility; ME: Metabolizable energy; WSC: Water-soluble carbohydrates; CP: Crude protein; NDF: Neutral detergent fibre; ADF: Acid detergent fibre; C: Cutting day; SS: Seed source/genotypes; DAS: Days after sowing.

The forage with the highest DMD was that produced with LBM1 and cut at day 65 (73%), while the lowest DMD was produced with forage grown using LBF1 and cut at day 210 (65%). Overall, DMD was higher (*P* < 0.001) for day 65 forage (72%) than for forage cut on day 110 (68%), day 150 (66%), day 180 (66%) or day 210 (65%); while the DMD of forage cut on day 110 was higher (*P* < 0.001) than that cut on day 210. DMD of Agaitti Berseem-2002 (68.3%) was higher (*P* < 0.05) than that from LBM1 (66.8%) but did not differ (*P* > 0.05) from that of LBF1 (67%). The maximum DOMD was produced by LBM1 when cut at day 65 (68%), while the minimum was produced by LBF1 when cut at day 210 (62%). Overall, DOMD was higher (*P* < 0.001) for day 65 forage (68%) than for forage cut on day 110 (64%), day 150 (63%), day 180 (63%) or day 210 (62%) whilst DOMD was higher (*P* < 0.001) with forage cut on day 110 than day 210. DOMD was higher (*P* < 0.05) for Agaitti Berseem-2002 forage (65%) compared to LBF1 (64%) or LBM1 (63%).

The ME was directly calculated from DOMD so the trend for ME was the same as for DOMD. The highest ME was from LBM1 cut at day 65 (10.8 MJ/kg DM), while the lowest was from LBF1 cut at day 210 (9.5 MJ/kg DM). In comparing forage cut at different days, ME was higher (*P* < 0.001) for day 65 forage (10.7 MJ/kg DM) than for forage cut on day 110 (10.0 MJ/kg DM), day 150 (9.77 MJ/kg DM), day 180 (9.79 MJ/ kg DM) or day 210 (9.57 MJ/kg DM), whilst ME was higher (*P* < 0.001) for forage cut on day 110 compared to day 210. Agaitti Berseem-2002 had higher ME (*P* < 0.05) of 10.13 MJ/kg DM than either LBF1 (9.93 MJ/kg DM) or LBM1 (9.87 MJ/kg DM).

LBF1 cut at day 65 had the highest CP content (29%) while LBM1 cut on day 210 had the lowest CP (23%). Forage cut at day 210 had the lowest (*P* < 0.001) CP content (23%) compared to all other forage cuts (25, 27, 38 and 28% for forage cut on days 180, 150, 110 and 65, respectively). Across the different genotypes, the CP content of forage cut at day 65 was higher (*P* < 0.001) than that of all other forage cuts, except that cut on day 110. The CP content of LBM1 (25.5%) was lower (*P* < 0.001) than LBF1 (26.4%) or Agaitti Berseem-2002 (26.8%). The CP levels of LBM1 and LBF1 fell as the berseem crops aged in contrast to Agaitti Berseem-2002 which retained relatively consistent CP levels until day 210.

LBM1 cut on day 180 had the highest NDF content (30.5%) while LBF1 cut on day 65 had the lowest NDF (25.5%). Forage cut on day 65 had the lowest (*P* < 0.001) NDF content (26.5%), whilst the NDF contents of forage cut at day 110 (30.2%) and day 150 (29.7%) were lower (*P* < 0.001) than when cut on days 180 (31.6%) and 210 (32.6%). The NDF content of Agaitti Berseem-2002 (29.2%) was lower (*P* < 0.001) than LBF1 (30.2%) or LBM1 (30.8%). LBM1 cut at day 65 had the lowest ADF content (18.8%) while LBF1 cut on day 210 had the highest (24.5%). Across the different genotypes, forage cut on day 65 had the lowest (*P* < 0.001) ADF content (19.2%) while that cut on day 210 had the highest (*P* < 0.001) (24.2%). The ADF content of Agaitti Berseem-2002 (21.3%) was lower (*P* < 0.001) than LBF1 (22%) or LBM1 (22.4%). Time of cutting but not genotype impacted on the ash content, with forage cutting at days 110 and 150 having higher (*P* < 0.001) ash content (15.2 and 15.1%, respectively) than forage cutting on days 65 (14.3%), 180 (14.4%) and 210 (14.4%). The forage quality parameters indicated that overall the Agaitti Berseem-2002 provided a better quality forage source than LBM1 and LBF1. Forage quality declined with later forage cuts, although, Agaitti Berseem-2002 retained forage quality for the longest duration (up to 210 days) with different cuttings during the entire growing season of the berseem crop.

### On-farm evaluation of recommended variety and cutting practices (Year 2 experiment)

Agaitti Berseem-2002 performed better than other genotypes in the 2012/13 growing season at all sites and again in 2013/14 on the UVAS site. Cutting regimens T2 and T3 were the best options to maximise forage (T3) and seed production (T2); therefore, these treatments were utilized in the second year trial.

#### Forage and seed yields parameters

Increasing the number of forage cuts (prior to seed harvest) from three to four, increased plant height, green forage yield and DM yield (*P* < 0.001) by 4.5%, 35%, and 45%, respectively (Table 3). Consistent with the previous growing season, the number of stems per square meter was not affected (*P* > 0.05) by the number of forage cuts prior to seed harvest. For seed yield parameters as the number of forage cuts prior to seed harvest increased from three to four, the average number of heads per square meter, number of seeds per head, 1000-seed weight and seed yield declined (*P* < 0.001) by 37%, 30%, 3%, and 57%, respectively (Table 3).

**Table 3:** The effect of different cutting treatments on forage and seed yield parameters of berseem clover grown using Agaitti Berseem-2002 on participating farmer field sites in the 2013–2014 growing season.

Parameter	Number of forage cuts prior to seed harvest		*P* value (±SED)
	T2 (3 Cuts)	T3 (4 Cuts)	
Number of stems per square meter	346.4	354.5	ns
Plant height (cm)	25.2^a^	26.4^b^	<0.001 ± 0.24
Green forage yield (t/ha)	48.2^a^	65.1^b^	<0.001 ± 1.74
Dry matter yield (t/ha)	6.1^a^	8.9^b^	<0.001 ± 0.27
Number of heads per square meter	534.7^b^	335.5^a^	<0.001 ± 23.19
Number of seeds per head	47.1^b^	33.15	<0.001 ± 2.40
1000-seed weight (g)	3.78^b^	3.58^a^	<0.001 ± 0.05
Predicted seed yield (kg/ha)	945.9^b^	410.8^a^	<0.001 ± 56.87

Values within rows with different superscripts vary significantly (P < 0.001).

#### Forage quality parameters

There were no significant (*P* > 0.05) differences between forage nutritive characteristics and cutting treatments (Table 4). Moreover, there was no direct linear relationship between the day at which forage was harvested and its nutritive value. Forage cut at day 180 had the highest (*P* < 0.001) DMD, DOMD, CP and WSC contents and the lowest (*P* < 0.001) NDF and ADF contents. It also had a higher (*P* < 0.001) ME than that of the forage cut on day 110 and 150. Forage cut at day 150 had the lowest (*P* < 0.001) DMD and DOMD.

**Table 4:** The effect of time of cutting (days) on forage quality parameters (dry matter basis) across cutting treatments grown using cv. Agaitti Berseem-2002 on participating farmer field sites in the 2013–2014 growing season.

Parameter	Time of forage cut (days after sowing)			
	65	110	150	180
Dry matter digestibility (%)	72.5^b^	73.0^b^	68.7^a^	74.9^c^
Digestible organic matter digestibility (%)	68.2^b^	68.7^b^	65.0^a^	70.3^c^
Metabolizable energy (MJ/kg)	10.46^bc^	10.35^ab^	10.30^a^	10.52^c^
Water-soluble carbohydrates (%)	2.79^a^	3.25^b^	2.79^a^	3.73^c^
Crude protein (%)	28.0^a^	28.7^a^	28.1^a^	31.5^b^
Neutral detergent fibre (%)	27.2^c^	25.8^b^	27.5^c^	22.9^a^
Acid detergent fibre (%)	19.2^c^	18.7^b^	21.5^c^	15.8^a^
Ash (%)	13.9^a^	14.52	14.7^b^	13.7^a^

Values within rows with different superscripts vary significantly (P < 0.001).

## Discussion

### Maximising forage and seed production

Both genotype and cutting treatments affected forage yield components. The Agaitti Berseem-2002 produced 39 and 46% higher green and DM yields, respectively compared to LBF1 (Table 1). These results are in agreement with the study of Ranjbar^20^, who reported an overall 21% increase in the DM yield with the use of an improved variety in Mazandaran province of Iran. Similarly, in a study in the Giza region of Egypt^21^, researchers reported a 48% increase in the green forage yield of berseem clover by using improved variety seed. Cutting management is the most crucial agronomic factor directly affecting yields for berseem clover^22^ and indirectly influences the nutritive value of the forage^23^. The forage yield; however, primarily depends on the phenological stage of growth when plants are cut. Frequent cutting increases the regeneration and growth rate of plants and therefore enhances green as well as DM yields^5^ as found in the present study. The increase in forage yield with cutting is likely associated with the source and sink relationship that exists within berseem plants. This relationship in forage crops such as berseem clover is very dynamic and changes with the growth of the plant which is greatly influenced by cutting management. Shoots are the main sink and the roots act as the main source of food reserves during the regrowth of plants after each cutting and this cycle of storage and reutilization of food reserves is repeated with every harvest. Frequent harvesting increases the regeneration and growth rate of berseem clover forage and therefore enhances forage and DM yields; however, premature (very early) cutting reduces food reserves and therefore reduces yields and plant survival^5^.

Lower DM yield increases were recorded at later cuttings with only a 36 and 43% increase (from T3 to T4) as compared to a 46 and 52% increase (from T2 to T3) in 2012/13 and 2013/14 growing seasons, respectively, from the earlier cuts in the present study. This was similar to previously reported 12–20% increase in DM in the first to third forage cuttings and a 53% increase during the fourth forage cut^24^. However, cutting at the sixth internode stage (30–35 cm height) proved to be the best cutting management practice to obtain maximum forage and seed yields^5^. As both cutting interval and the height to which forage is cut can impact on plant biomass, cutting intervals and height were kept constant in the present study. The major cause of enhancement of berseem clover forage productivity was associated with plant height and early stages of plant growth^20^. Similarly, plant height decreased post-harvest when taking forage cuttings at the sixth internode elongation and at early flowering^25^. However, post-harvest plant height increased when plants were at the stage of physiological seed maturity^9^. In addition to this, the amount of biomass and leaf area produced was less when berseem clover was cut to a 3 cm height as compared to 6 cm, and that higher DM yields were obtained with a shorter cutting interval (days) at 6 cm height compared with 3 cm with longer cutting intervals^26^.

As shown in Fig. 2, the Agaitti Berseem-2002 outperformed both the LBM1 and LBF1 sources in all parameters on every occasion (across all sites/farms). The variety’s enhanced vigour, as demonstrated by its larger seed size^27^, was likely to be a major factor affecting this result. Moreover, low stem density may be attributed to low seed vigour of different berseem clover varieties^24^. Thus, the significantly greater stem density within the Agaitti Berseem-2002 treatments is likely to have been influenced by increased plant vigour and has led to the greater mass of forage and seed produced. Cutting treatment/frequency had no effect (*P* > 0.05) on the number of stems present at forage harvest indicating that in this experiment the final number of stems was determined prior to 65 days and not adversely affected by cutting. Considerable variation has been found between berseem clover genotypes in terms of their plant growth parameters (primarily plant height), and forage and seed yields which are the ultimate expression of genotype productivity associated with physiological mechanisms that occur throughout the life cycle within an environment^28^. In contrast with the present study, berseem clover produced a significantly higher number of stems when cut at 60–70 DAS (first cut) compared to 150 (last cut) DAS^7^. Frequent forage cuttings resulted in higher forage and seed yields due to an increase in stemming, regeneration and growth rate of berseem plants^5^. However, differences in stem numbers and plant heights ultimately influence the green forage and DM yields of berseem clover^24^. At later growth stages high temperatures can negatively impact on plant growth and development, resulting in a lower number of stems per unit area, lower plant height and ultimately a reduction in forage and seed yields^7^, which were similar to the results of the present study as both forage and seed yields were reduced at later cuttings. However, overall forage production increased due to additional forage cut (Table 1).

In all genotypes and years of this experiment, cumulative dry forage biomass levels increased with each subsequent cut, with the highest DM yield being achieved with Agaitti Berseem-2002 (13.4 t/ha), which was 46% higher than LBF1 and 30% higher than LBM1. This is consistent with the findings of a significant increase in DM forage yield which occurred with the addition of every forage cut of berseem clover in an Indian trial^7^. Similarly, a 26% increase in DM production was also reported by using improved cultivars^20^. Moreover, this indicates that the improved forage varieties have greater yield potential throughout the growing season in different agro-climatic conditions. These results are in agreement with the findings of different researchers around the world in obtaining higher DM yields with the use of improved cultivars of berseem clover^7,9,26^. The possible reason for the increase in DM yield was a rise in ambient temperatures (increased DM partitioning) during the months of February to April after the winter months of December and January (Fig. 1). Clover growth rates are temperature dependent, with low growth rates observed during cold or hot conditions. Periodic yields between T1 and T2, T2 and T3 and then T3 and T4 were the same for LBF1 and LBM1 but varied for Agaitti Berseem-2002 (Table 1). This suggests the landrace genotypes were less responsive to favourable conditions like soil moisture and temperature than improved variety (Agaitti Berseem-2002).

The longer the vegetative period, the greater will be the green forage and DM yields of berseem clover^7^. However, after the third forage cut the plant regrowth is reduced because of low soil moisture and a rapid shift to the reproductive stage^24^ as found in the present study. Moreover, the high temperatures at later growth stages can negatively affect the growth and development of plants and resulted in a lower number of stems per unit area and lower plant height leading to a reduction in forage and seed yields^7^. In the present study, the stem number of Agaitti Berseem-2002 declined after T1 but recovered to similar numbers at T4. The initial decline may have been due to cold temperature, however, the recovery in stem numbers at T4 was due to favourable growing conditions during the final period. Conversely stem numbers remained constant throughout the growing season for both landraces (LBF1 and LBM1), suggesting that these genotypes were more tolerant of higher temperatures, possible stress or some other environmental factors at that time. It also suggests that these landrace cultivars may contain some genetic advantages for future incorporation into breeding programs. Plant populations of berseem clover decreased linearly as the growing season progressed regardless of the frequency of cuttings and cutting height of plants^26^. Moreover, plant mortality was higher with low stubble heights due to depletion of root reserves, making growth/recovery time and cutting height important factors contributing to stem densities and ultimately yields of berseem clover.

Seed production in berseem clover represents a strong inverse source-sink relationship between vegetative (leaves and stems) and reproductive (flowers) plant organs. Seed acts as the sink for photosynthates from vegetative plant organs as the source of food reserves translocated to seed filling during reproductive growth^5^. Thus, a strong relationship is expected between forage and seed yields as was observed in the present study. The frequency of cutting^9^ and the timing of the last forage cut^7^ have been shown to affect the distribution and amount of forage DM, subsequently impacting on both forage and seed yields, similar to the findings which were identified in the present study as shown in Table 1. Seed yields varied between farms (Fig. 3) which may have been due to differences in environmental conditions such as temperature and rainfall, soil conditions and water quality of different regions as well as the management skills of the farmers. The variation between farms indicates that there may have been differences in management ability of the farmers so benefits for the broader population would likely be even more variable. Regardless, the profit margins (from both forage and seed) would still be greater from using Agaitti Berseem-2002 in comparison to the local landraces (like LBF1 and LBM1). This would be a consequence of the higher genetic capability and phenotypic potential of the improved variety being translated into better quality forage and higher seed yields.

Increasing seed production is normally at the expense of forage yield and therefore management of cutting time is important in balancing forage and seed yields of berseem clover^7,29^. How the management of this trade-off is undertaken will depend on the importance of forage or seed production to the farming systems. Hence, two cutting frequencies were chosen for the second year of study in this research experiment, as the T2 (three forage cuts) and T3 treatments (four forage cuts) favoured either seed or forage production, respectively (Fig. 4). Berseem clover seed yield was enhanced through cutting treatments (up to three to four forage cuts) in the present study, which is in agreement with previous studies in Italy^5^ and Australia^29^. These studies had reported higher seed yields of berseem clover obtained during regrowth after forage cuttings. However, this needs to be balanced to ensure the availability of carbohydrates and photosynthates for regeneration of productive stems and seed development.

Increasing the number of forage cuttings can adversely affect the regeneration ability of the plant, resulting in a reduction in the potential of floral buds and ultimately reduced seed yield^5^. A lower number of forage cuts can potentially increase translocation of food reserves to the heads resulting in greater seed production^7^. Such an effect was found in the present study (Table 1) for T4 where predicted seed yield averaged only 84 kg/ha across the genotypes. Dry and hot weather conditions during the reproductive stage will result in early maturity of the seed crop, poor fertilization (due to pollen death) and thus a significant reduction in seed yield^7^. The paucity of seed produced by the T4 treatment across all genotypes was likely a result of a combination of reduced plant biomass and excessively hot conditions for seed setting and development. Taking a systems view, more frequent cutting of berseem clover is an effective method of reducing weed populations (up to 80%) by increasing stem density with less light penetration^30^. This also leads to more uniform flowering, resulting in higher seed yields^9,31^. Thus, farmers need to weigh-up these competing factors in managing their berseem crops.

The temperature and relative humidity conditions experienced at the time of flowering in the present study (Fig. 1) were unfavourable for pollination resulting in a significant reduction in seed yields of cutting treatments T3 and T4 (Table 1) compared to T2 when environmental conditions were more favourable for pollination. These findings were in line with the studies that reported temperatures of 28–32 °C with a relative humidity of 45–55%, which were favourable for maximum pollination in berseem clover crops^32,33^. The timing of the last forage cut prior to seed harvesting can also impact on the efficiency of pollinators, namely honeybees. High temperatures and low relative humidity during flowering can greatly reduce the efficiency of pollinators. Thus, forage cuts that delay flowering until later in the season may reduce the efficiency of pollination and thereby seed production.

The length of the reproductive phase (from flower initiation to crop maturity or seed harvest) is very important in berseem clover seed production and is significantly affected by forage cutting. Predicted seed yields were highest for T2 and lowest for T4 treatments. This is in agreement with Lowe and Bowdler^29^, who found there was a significant inverse relationship between seed yield and the timing of the last forage cut. Berseem clover had a longer reproductive phase of 33–36 days (achieved when the last forage cut was taken 150 DAS), and produced maximum seed yields of 443 kg/ha as compared to a shorter reproductive phase of 21–27 days (when cut 170 DAS) producing only 315 kg seed/ha in Hisar, India^7^. There was no difference in seed yield when the last forage cuts were at 150 DAS (T2) and 180 DAS (T3) in the present study although major seed yield reductions occurred if delayed till 210 DAS (T4). Using Agaitti Berseem-2002 maximised both forage and seed production resulting in a 46% increase in DM forage production and a 211% increase in seed production and hence was identified as the best genotype for the second year of research trials (evaluation trials) in the present study. Similar results of selecting the appropriate genotype were shown to maximise both forage and seed production of berseem clover^8,22^.

### Maximising forage quality

Forage optimization in livestock feeding requires an understanding of DM partitioning, plant composition and the changes which occurred in the nutritive values of forage by different cutting practices. Berseem forage quality was generally high across all forage cuts collected during the experiment, though variation did exist. In the present study, the forage with the highest nutritive value (DMD, DOMD, ME and CP) was that cut at days 65 and 110 (Table 2) and is likely due to favourable growing conditions (Fig. 1) in the second growing season. The nutritive value of berseem forage is influenced by growing season, growth stage and time of cutting^23^, and soil fertility and soil moisture availability^7,34^. Growing season, soil nutrition and soil moisture availability were not directly tested in the present study, although differences between seasons 1 and 2 at UVAS demonstrated that with increased rainfall in the second season (thereby influencing growing season length and soil moisture particularly), the forage quality parameters improved. Growth stage in combination with time of cutting would have greatly been influenced by genotype. Cutting times, though set according to the number of days, would have varied with genotype maturity. Smallholder dairy production in Pakistan is limited not only by the availability of forage but also its quality, with digestibility, ME and CP likely to be the most limiting factors. The recommended forage harvesting regimen for berseem clover is that it should be first cut 60–70 DAS with subsequent cuttings at 30–40 days interval in order to obtain maximum and high nutritive value forage^7,35^, as practiced in the present study. To maximise the nutritive value of the forage it is recommended that it can be cut at the fourth internode^36^. The leaves are of higher nutritive value than stems^23^ and thus later cuttings are of lower nutritive value due to an increase in the proportion of stem material.

Plant maturity is the major factor contributing to changes in forage digestibility^37^. Hence, the interaction between cutting day and genotype which both influence the rate of plant maturity, affected forage quality across all measured parameters with the exception of water-soluble carbohydrates and ash (Table 2). While later forage cuts typically have greater DM yields, digestibility is lower due to a reduction in the leaf to stem ratio and an increase in stem proportion (fibre content) with successive harvests^23,37^. While increasing the frequency of defoliation decreases DM yield of berseem clover, it has been shown to increase forage quality, predominantly as a consequence of an increase in the leaf to stem ratio^5^. The frequent cutting of berseem clover produces high leaf contents, consequently increasing the leaf to stem ratio, resulting in higher quality forage^26,36^. Similarly, late forage cutting at early flowering increased the total DM yield with lower DOMD and CP values^5^. However, not all researchers agree that the later forage cuts give greater DM yields. In a study conducted in Italy, an inverse relationship was found between stage of growth at cutting and DM yield in the subsequent cuttings of berseem clover^38^.

The forage yield of berseem clover forage was negatively correlated with CP, with forage cut at an earlier growth stage having lower yield but greater CP contents due to plant maturity^36^. This is consistent with other species and is similar to the results of the present study. Frequent forage cuttings resulted in an increase in CP content^39^ which was not the case in the present study where forage cut at day 210 had the lowest (*P* < 0.001) CP content. However, this is in agreement with the findings of other researchers who found a decline in the CP and DMD of berseem clover plants with progress through the growing season (winter > spring > summer)^23^. The authors speculate this is due to higher temperatures during the growing period which increased cell wall and lignin contents thus reducing digestibility, and faster growth resulting in plants being more physiologically mature at later harvests, which reduced both DMD and CP contents^34^. Overall, the quality of forage grown using Agaitti Berseem-2002 was higher than that grown using either LBF1 or LBM1, although CP content did not vary (*P* > 0.05) from that of forage grown using LBF1. Therefore, to maximise forage production (quantity and quality) and seed production, the use of Agaitti Berseem-2002 with either three (day 65, 110 and 150) or four (day 65, 110, 150 and 180) forage cuts prior to seed harvest is recommended.

### On-farm evaluation of recommended variety and cutting practices

Conventional crop breeding has increased the profitability of large farmers in Pakistan; however, smallholder farmers have shown little interest in adoption due to a lack of resources and availability of crop inputs^2^. This has resulted in crop failure, low yields, malnutrition and of course poverty^40^. Farmers participating in varietal selection and evaluation provides an alternative approach to overcome many small farm limitations to adoption^41^ and empowers smallholder farmers to have a say in deciding plant characteristics best suited to their needs and environment. Farmer knowledge on how best to grow forages varies widely and has resulted in wide variation in forage productivity. The variation in the forage productivity may, however, be due to seed quality, farming practices, soils and irrigation water used^2^.

Forage seed production at the farm level (and its retention for future sowings) is of great importance to farm productivity and profitability, and is also helpful in genetic improvement through the selection of productive forage varieties. It was evident from a recent study that the addition of seed yield parameters amongst the forage variety selection criteria resulted in the selection of the best genotypes instead of selection being made only on the basis of DM yield^6^, which is not a preferred selection trait of many farmers^8^. In recent studies, researchers have found that improved genotypes of berseem clover selected for seed production alone or in combination with other traits gave greater forage and seed yields compared to local and conventional genotypes/landraces^8,28^. Moreover, they found that the Italian genotypes produced a greater DM yield and were more adaptable to the environments than Egyptian genotypes in Sardinia, Italy. However, Egyptian genotypes produced greater seed yields^10^. Therefore, it is important that any improved forage varieties are able to not only produce high seed yields but also produce maximum forage of better quality in order to ensure adoption^2,42^.

The Agaitti Berseem-2002 variety performed well for both seed and forage production and so is likely to be favoured by farmers. Selection of genotypes with early seed set were favourable for high temperature and low relative humidity areas^33^. These were linked with the regulation of the movement of pollinators, which set the genotypes for early blooming and high pollination efficiency and thus increased seed yield of berseem clover. The improved variety (Agaitti Berseem-2002) used in the present study was best suited to the environment and farming conditions both in terms of productivity as well as early seed set, and thus fits well with the current cropping system of the study areas (Kasur and Okara districts of Punjab, Pakistan).

Varietal selection and evaluation using a farmer participatory research approach appeared to work in the present study. Farmers responded positively to the improved Agaitti Berseem-2002 variety when evaluated against the local varieties/landraces (LBF1 and LBM1) which performed poorly (Table 1). Farmers were eager to test the new improved variety and were extremely critical of the inclusion of unproductive varieties. Although the research design was partly compromised in the second year due to the reduction of treatments on the farmer field sites, the consistency of results coming from the UVAS research site proved this was not detrimental to the research outcomes. Further, the reproduction of the performance of treatments (Agaitti Berseem-2002, T2 and T3) carried out in the second year at the farm sites would have greatly enhanced surrounding farmer perceptions of the variety and management technologies displayed whilst supporting the participant farmers financially. Thus, a second outcome was achieved by making this small compromise in research design. Working with the farming community, introduced the concept of farmer-based seed production at the village level and was demonstrated to be of great importance for farmer acceptance and adoption, and provided a sustainable way to improve forage and seed shortage problems through increased productivity as alluded to in this paper.

## Conclusion

The farmers’ varietal selection, evaluation and cutting management studies of the conventional and improved varieties of berseem clover in Kasur and Okara districts showed a wide variation in green forage, DM and seed yields both for total seasonal production and its distribution throughout the growing season. The Agaitti Berseem-2002 (improved variety) produced the highest weight of green forage, DM and seed yields of better quality across all farm sites, particularly when three forage cuts at 65, 110 and 150 DAS were taken prior to seed harvest. The use of Agaitti Berseem-2002 in smallholder farming systems has been shown to increase forage DM (46%) as well as seed (211%) production at the farm level. These dramatic production gains will not only help reduce on-farm feed deficits but also has the potential to be a catalyst for the establishment of new markets for both the surplus forage and seed. Moreover, the application of knowledge in technology development increases smallholder farmers’ productivity and income while enhancing their livelihoods and thus helping in poverty reduction.

## References

[1] Dost, M., Misri, B., EL-Nahrawy, M., Khan, S. & Serkan, A. *Egyptian Clover (Trifolium alexandrinum); King of Forage Crops*. Cairo: Food and Agriculture Organization of the United Nations (2014).

[2] Tufail MS (2017). Constraints to adoption of improved technology for berseem clover (Trifolium alexandrinum L.) cultivation in Punjab, Pakistan. Experimental Agriculture.

[3] David S (2004). Farmer seed enterprises: a sustainable approach to seed delivery?. Agriculture and Human Values.

[4] Anwar MZ (2012). Small farmers perceptions regarding improved fodder and forage varieties: results of participatory on farm research. Pakistan Journal of Agricultural Research.

[5] Iannucci A (2001). Effects of harvest management on growth dynamics, forage and seed yield in berseem clover. European Journal of Agronomy.

[6] Pecetti L, Usai R, Romani M, Fraschini P, Salis M (2012). Evaluation of berseem clover (Trifolium alexandrinum L.) germplasm in Sardinia, Italy. Italian. Journal of Agronomy.

[7] Sardana V, Narwal SS (2000). Influence of time of sowing and last cut for fodder on the fodder and seed yields of Egyptian clover. Journal of Agricultural Science.

[8] Iannucci A, Annicchiarico P (2011). Seed and forage yield of advanced generation synthetics of berseem clover derived from partly inbred parents under different harvesting regimes. Plant Breeding.

[9] Amato G, Giambalvo D, Ruisi P (2013). Cut and post-cut herbage management affects berseem clover seed yield. Agronomy Journal.

[10] Tufail MS, Krebs GL, Southwell A, Wynn PC (2018). Village-based forage seed enterprises: A sustainable intervention for rural development in the mixed farming systems of Pakistan. Australasian Agribusiness Review.

[11] Morris M, Bellon M (2004). Participatory plant breeding research: opportunities and challenges for the international crop improvement system. Euphytica.

[12] Qamar-uz-Zaman C, Rasul G (2004). Agro-climatic classification of Pakistan. Pakistan Journal of Meteorology.

[13] Tufail MS (2018). The effect of rhizobium seed inoculation on yields and quality of forage and seed of berseem clover (Trifolium alexandrinum L.) and its impact on soil fertility and smallholder farmer’s income. The Journal of Animal and Plant Sciences.

[14] Van Soest PJV, Robertson JB, Lewis BA (1991). Methods for dietary fiber, neutral detergent fiber, and nonstarch polysaccharides in relation to animal nutrition. Journal of Dairy Science.

[15] AFIA. Laboratory Methods Manual; A Reference Manual of Standard Methods for Analysis of Fodder. 8 edn, Vol. 8, Australian Fodder Industry Association Inc. (2014).

[16] Galyean, M. L. Laboratory Procedures in Animal Nutrition Research. Department of Animal and Food Sciences, Texas Tech University, Lubbock, United States (2010).

[17] Tufail MS (2019). Seeding rate effects on yield components and forage quality of Agaitti Berseem-2002 – an improved variety of berseem clover. Journal of Crop Improvement.

[18] GenStat Reference Manual (Release 17) v. 17th VSN International, Hemel Hempstead, UK (2014).

[19] Government of Pakistan. Climate data processing centre, www.pmd.gov.pk/cdpc/home.htm (2015).

[20] Ranjbar GA (2007). Forage and hay yield performance of different berseem clover (Trifolium alexandinum L.) genotypes in Mazandaran conditions. Asian Journal of Plant Sciences.

[21] Radwan, M. S., Abdel, K. I. & El-Zanaty, R. I. Factors affecting the productivity of berseem clover in Egypt, www.researchgate.net/publication/235910786 (2006).

[22] Martiniello P (1998). Influence of agronomic factors on the relationship between forage production and seed yield in perennial forage grasses and legumes in a mediterranean environment. Agronomie.

[23] De Santis G, Iannucci A, Dantone D, Chiaravalle E (2004). Changes during growth in the nutritive value of components of berseem clover (Trifolium alexandrinum L.) under different cutting treatments in a mediterranean region. Grass and Forage Science.

[24] Amato, G., Giambalvo, D., Giorgio, G. D. & Graziano, D. Performance of berseem clover varieties grown in organic farming in a semi-arid mediterranean environment. *Breeding and variety progress for conventional and organic agriculture*, 150–153 (2007).

[25] Iannucci A, Rascio A, Russo M, Di Fonzo N, Martiniello P (2000). Physiological responses to water stress following a conditioning period in berseem clover. Plant and Soil Journal.

[26] Giambalvo D, Amato G, Stringi L (2011). Effects of stubble height and cutting frequency on regrowth of Berseem clover in a mediterranean semiarid environment. Crop Science.

[27] Tufail, M. S. Development of berseem clover (*Trifolium alexandrinum L*.), village-based forage seed enterprises for the profitability and sustainability of smallholder farmers of Pakistan in mixed farming systems. Doctor of Philosophy thesis, Charles Sturt University (2016).

[28] Iannucci A (2004). Effect of generation of inbreeding, cutting treatment and year on agronomic traits in berseem populations. Euphytica.

[29] Lowe KF, Bowdler TM (1982). Note on the effect of sowing date, defoliation management and closure date on the seed production and forage yield of berseem clover (Trifolium alexandrinum). Agricultural Research.

[30] Pathan SH, Kamble AB, Gavit MG (2013). Integrated weed management in berseem. Indian Journal of Weed Science.

[31] Pasumarty SV, Higuchi S, Murata T (1996). Influence of seed quality on field establishment and forage yield of white clover (Trifolium repens L.). Journal of Agronomy and Crop Science.

[32] Ismail A-HM, Owayss AA, Mohanny KM, Salem RA (2013). Evaluation of pollen collected by honey bee (Apis mellifera L.) colonies at Fayoum Governorate, Egypt. Part 1: Botanical origin. Journal of the Saudi Society of Agricultural Sciences.

[33] El-Naby A, Zeinab M, Sakr HO (2012). Influence of ecological factors on seed setting and fertility of five Egyptian clover (Trifolium alexandrinum L.) cultivars. Asian Journal of Plant Science and Research.

[34] Fulkerson WJ (2007). Nutritive value of forage species grown in the warm temperate climate of Australia for dairy cows: grasses and legumes. Livestock Science.

[35] Oushy, H. *Fact Sheet: Berseem Clover*. College of Agricultural, Consumer, and Environmental Sciences, New Mexico State University, USA (2008).

[36] Iannucci A, Fonzo ND, Martiniello P (1996). Effects of the developmental stage at harvest on dry matter and chemical component partitioning in berseem. Journal of Agronomy and Crop Science.

[37] Laghari HH, Channa AD, Solangi AA, Soomro SA (2000). Comparative digestibility of different cuts of berseem (Trifolium alexandrinum) in sheep. Pakistan Journal of Biological Sciences.

[38] Martiniello P, De Santis G, Iannucci A (1996). Effect of phenological stages on plant dry matter partitioning and seed production in berseem (Trifolium alexandrinum L.). Journal of Agronomy and Crop Science.

[39] Kandil AA, Salama AM, El-Moursy SA, Abido WA (2005). Productivity of Egyptian clover as affected by seeding rates and cutting schedules II-Chemical dry matter analysis. Pakistan Journal of Biological Sciences.

[40] Ceccarelli S, Grando S (2007). Decentralized-participatory plant breeding: an example of demand driven research. Euphytica.

[41] Almekinders CJM, Elings A (2001). Collaboration of farmers and breeders: Participatory crop improvement in perspective. Euphytica.

[42] Iannucci A, Martiniello P (1998). Analysis of seed yield components in four mediterranean annual clovers. Field Crops Research.

